# Supplementary material for: Observation of spin-glass-like characteristics in ferrimagnetic TbCo through energy-level-selective approach
Source: Nat Commun. 2022 Sep 21;13:5530. doi: 10.1038/s41467-022-33195-y (PMC9492764; doi:10.1038/s41467-022-33195-y)
Supplement: Supplementary file 1 — Supplementary Information [file 41467_2022_33195_MOESM1_ESM.pdf]

Supplementary Notes for

"Observation of spin-glass-like characteristics in ferrimagnetic TbCo through energy-level-selective approach"

Ji-Ho Park[1†], Won Tae Kim[1†], Woonjae Won[1], Jun-Ho Kang[1], Soogil Lee[2], Byong-Guk Park[2], Byoung S. Ham[3], Younghun Jo[4], Fabian. Rotermund[1★] & Kab-Jin Kim[1★]

[1]*Department of Physics, Korea Advanced Institute of Science and Technology (KAIST), Daejeon, Republic of Korea*

[2]*Department of Materials Science and Engineering and KI for Nanocentury, KAIST, Daejeon 34141, Republic of Korea*

[3]*School of Electrical Engineering and Computer Science, GIST, Gwangju 61005, Republic of Korea*

[4]*Center for Scientific Instrumentation, KBSI, Daejeon 34133, South Korea*

-Contents-

Note 1. Temperature-dependent Hall measurement of TbCo film

Note 2. Partial magnetization switching at film edge

Note 3. Background subtraction of VSM measurement

Note 4. The relation between Kerr signal and magnetic moment at different energy levels

Note 5. Comparability between optical and Hall and VSM measurements

Note 6. Polar MOKE results of TbCo/Pt with 400-nm and 800-nm laser excitation at room temperature

Note 7. Quasi-static measurement of slow spin dynamics in TbCo

Note 8. THz wave emission through inverse spin Hall effect

Note 9. THz Emission and VSM results for in-plane moment

Note 10. Raw THz emission signals from TbCo/Pt and Co/Pt after field cooling

Note 11. Measurement of random anisotropy of Tb

Note 12. Spin-glass-like slow dynamics in GdFeCo

Note 13. Experimental setup for THz emission spectroscopy, static polar MOKE and TR-MOKE

Note 1. Temperature-dependent Hall measurement of TbCo film

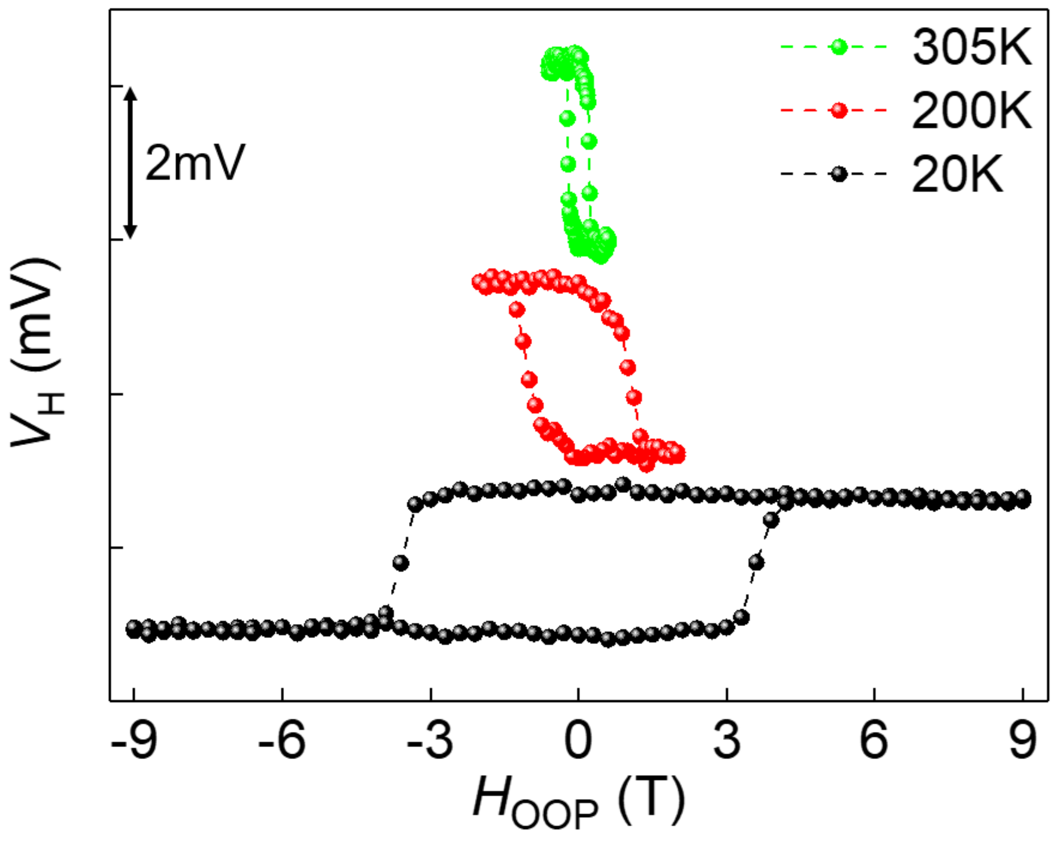

Fig. S1. Anomalous Hall effect voltage of TbCo film as a function of out of plane field (H_{OOP}) for different temperatures.

Fig. S1 shows the Hall measurement results of Ta (1.5 nm)/Pt (5 nm)/$Tb_{25}Co_{75}$ (20 nm)/Ta (1.5 nm) film on MgO substrate at different temperatures. The square hysteresis loop for H_{OOP} indicates the perpendicular magnetic anisotropy (PMA) of TbCo film for all measured temperatures. The clockwise hysteresis loop is observed at T = 305 and 200 K, while the sign of hysteresis is reversed to the counter-clockwise at T = 20 K. This indicates that the m_{co} is dominant over the m_{Tb} at T = 305 and 200 K, while the m_{Tb} becomes dominant at T = 20 K, showing a typical feature of RE-TM ferrimagnet across the magnetization compensation point. The coercive field increases with decreasing temperature, which is consistent with the previous work [1].

Note 2. Partial magnetization switching at film edge

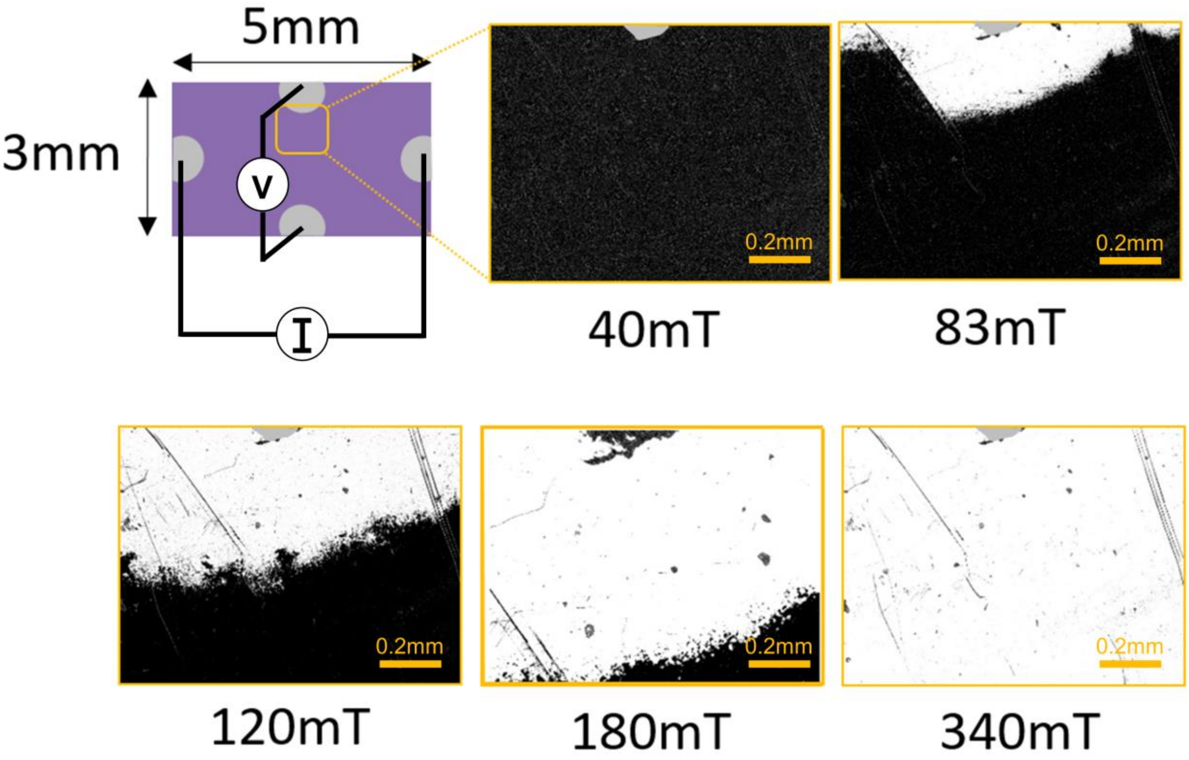

Fig. S2. Magnetic domain nucleation and expansion in TbCo film observed by wide field MOKE microscope.

As can be seen in Figs. 2(c) and 2(g) of main text, there is a small step in the hysteresis near H_{OOP} ~ ±50 mT, which we ascribed to the partial magnetization switching at edges. To confirm this, we checked the magnetization switching of TbCo film using magneto-optical Kerr effect (MOKE) microscopy. Figure S2 shows the magnetization switching procedure near the upper electrode contact. As we increase the magnetic field, the magnetic domain is firstly nucleated around H_{OOP} ~ 40 mT and subsequently expanded slightly with increasing the magnetic field. The magnetization of the film is eventually switched around H_{OOP} ~ 340 mT. This observation is consistent with our observation shown in Figs. 2(c) and 2(g). We note that the

different step heights in Fig. (c) and Fig. 2(g) are due to the different measurement techniques; Hall measurement (Fig. 2(c)) is sensitive to the region where the current flows and thereby can measure the Hall signal weighted by current density of each region, while the VSM measurement (Fig. 2(g)) detects the magnetization of total volume of sample without weighting.

Note 3. Background subtraction of VSM measurement

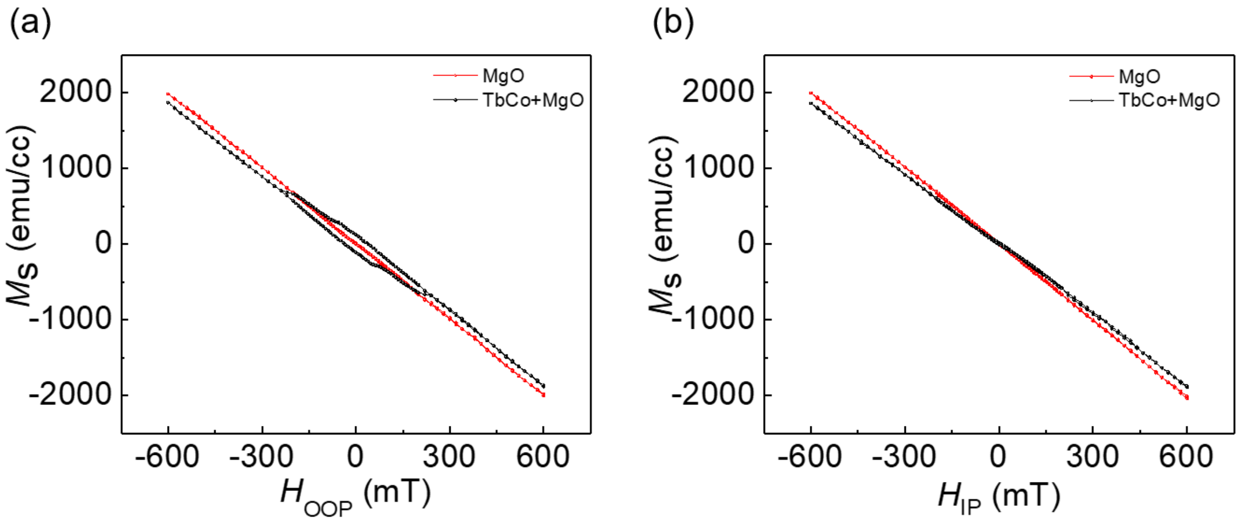

Fig. S3. VSM results under (a) out of plane field sweep and (b) in plane field sweep. Black (red) data shows the result for TbCo/substrate (substrate-only).

Generally, the linear background in VSM data originates from the diamagnetic response of the substrate. Therefore, one can simply remove it by subtracting the linear component from the measured VSM curve. In our measurement, however, this subtraction process is inappropriate because the unsaturated magnetic moment could give the linear signal in VSM data. To overcome this difficulty, we have devised a more accurate background subtraction process. We prepared two samples: one is the bare substrate and the other is the TbCo/substrate. Here, the substrates for two samples are almost identical with each other (thickness, width and length differences between two substrates were less than 1%). We then performed the VSM measurement for two samples using the same experimental protocol. Finally, we subtracted the signal of bare substrate from that of TbCo/substrate. Figures S3(a) and S3(b) show the raw VSM signals for OOP (a) and IP (b) for TbCo/substrate (black) and bare substrate (red). For OOP signal (S3(a)), the linear component from the TbCo/substrate and bare substrate has same slope, resulting in an almost perfect background subtraction, as shown in Fig. 2(g) of main text. However, for IP signal (S3(b)), the slopes for two samples are different and thus, the linear component cannot be completely removed, as shown in Fig. 2(i) of main text. This means that the remained linear slope in Fig. 2(i) comes from the TbCo itself.

Note 4. The relation between Kerr signal and magnetic moment at different energy levels

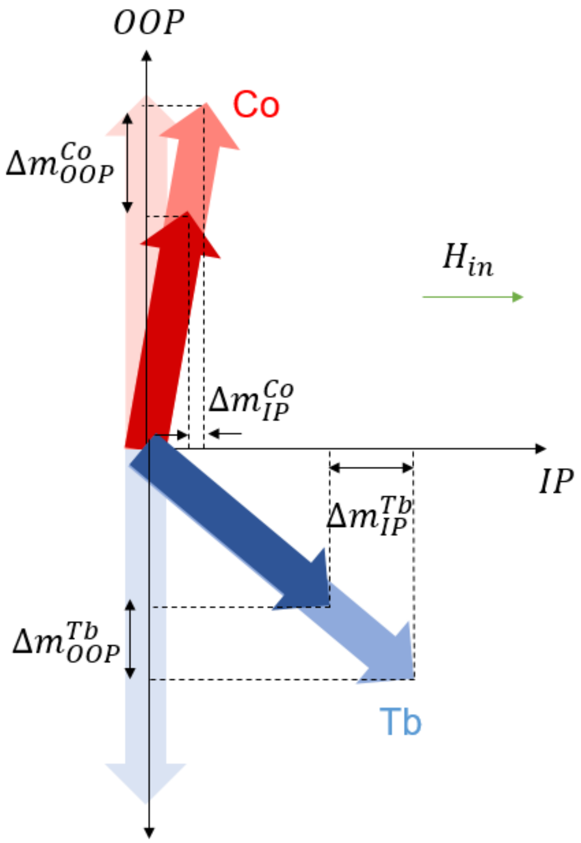

Fig. S4. Schematic illustration of magnetic moment configurations of Co and Tb in ferrimagnetic TbCo under in-plane magnetic field.

In this section, we explain the relation between the Kerr signal and magnetic moment in a more quantitative way. Figure S4 shows the schematic illustration of magnetic moment configurations of Co and Tb in ferrimagnetic TbCo under in-plane magnetic field. Here, Co and Tb directed in opposite direction along out-of-plane (OOP) direction due to antiparallel exchange interaction, while they are both tilted along the same in-plane (IP) direction due to presence of in-plane magnetic field. Since the TR-MOKE measure the laser-driven ultrafast demagnetization process, the measured variation of Kerr signal represents the amount of magnetic moment reduction, $\Delta m = \eta m$, where m is the magnetic moment and η is the demagnetization efficiency. We assume that the variation of Kerr signal, $\Delta\theta$, is proportional to that of

magnetic moment Δm, as it is generally accepted in the community.

We first check the relation of $\Delta\theta_K^{IP}/\Delta\theta_K^{OOP}$ with the magnetic moments near the Fermi energy level. In TbCo, the magnetic moment near the Fermi level is largely dominated by the Co 3d moment. Therefore, the Kerr signal ratio $\Delta\theta_K^{IP}/\Delta\theta_K^{OOP}$ can be expressed by

$$\frac{\Delta\theta_K^{IP}}{\Delta\theta_K^{OOP}} \sim \frac{\Delta m_{IP}^{Co}}{\Delta m_{OOP}^{Co}} = \frac{\Delta m^{Co} sin\varphi_{Co}}{\Delta m^{Co} cos\varphi_{Co}} = tan\varphi_{Co}, \qquad \text{(S1)}$$

where φ_{Co} is the tilting angle of Co moment by the in-plane magnetic field. This indicates that the Kerr signal ratio between IP and OOP direction near Fermi level is mainly governed by the tilting angle of Co, which approximately explains the TR-MOKE results for $\lambda = 800$ nm (here, 'approximately' means that the laser with $\lambda = 800$ nm can probe not only the Fermi level but also the deeper energy level down to $E_{\lambda=800nm} =$ 1.55 eV).

Unlike the magnetic moment near Fermi level, the magnetic moments at deeper energy level is more affected by the Tb moment, because the 4f level of Tb lies at deeper energy level. Therefore, if we use a laser of $\lambda = 400$ nm ($E_{\lambda=400nm} =$ 3.1 eV), it can probe not only the Co moment near Fermi level but also the Tb moment at deeper energy level. Then, the Kerr signal ratio $\Delta\theta_K^{IP}/\Delta\theta_K^{OOP}$ can be expressed by

$$\frac{\Delta\theta_K^{IP}}{\Delta\theta_K^{OOP}} \sim \frac{\Delta m_{IP}^{Co}+\Delta m_{IP}^{Tb}}{\Delta m_{OOP}^{Co}-\Delta m_{OOP}^{Tb}} = \frac{\Delta m^{Co} sin\varphi_{Co}+\Delta m^{Tb} sin\varphi_{Tb}}{\Delta m^{Co} cos\varphi_{Co}-\Delta m^{Tb} cos\varphi_{Tb}}. \qquad \text{(S2)}$$

Here, φ_{Tb} is the tilting angle of Tb moment. We note that the plus (minus) sign at numerator (denominator) indicates that the magnetic moments of Co and Tb align along the same (opposite) direction for IP (OOP) direction. This relation explains the TR-MOKE results for $\lambda = 400$ nm.

To be more quantitative, we roughly estimate the in-plane field-driven tilting angle of each magnetic moment using above equations. According to Fig. 3(d) in the main text, the $\Delta\theta_K^{IP}/\Delta\theta_K^{OOP} \sim$ 0.1 for $\lambda = 800$ nm (orange symbols in Fig. 3(d)). Therefore, the tilting angle of Co moment is approximately $\varphi_{Co} =$ 6° based on Eq. (S1). On the other hand, $\Delta\theta_K^{IP}/\Delta\theta_K^{OOP} \sim 10$ for $\lambda = 400$ nm (blue symbols in Fig.

3(d)). If we consider that $\Delta m^{Co} \sim \Delta m^{Tb}$ because the composition of our sample ($Co_{75}Tb_{25}$) is near the compensation point, then the tilting angle of Tb is approximately $\varphi_{Tb} = 17°$ based on Eq. (S2). Therefore, the different Kerr signal ratio of $\Delta\theta_K^{IP}/\Delta\theta_K^{OOP}$ for different wavelength manifests that the Tb moment, which lies at deeper energy level, is more susceptible to the in-plane magnetic field.

Note 5. Comparability between optical and Hall and VSM measurements

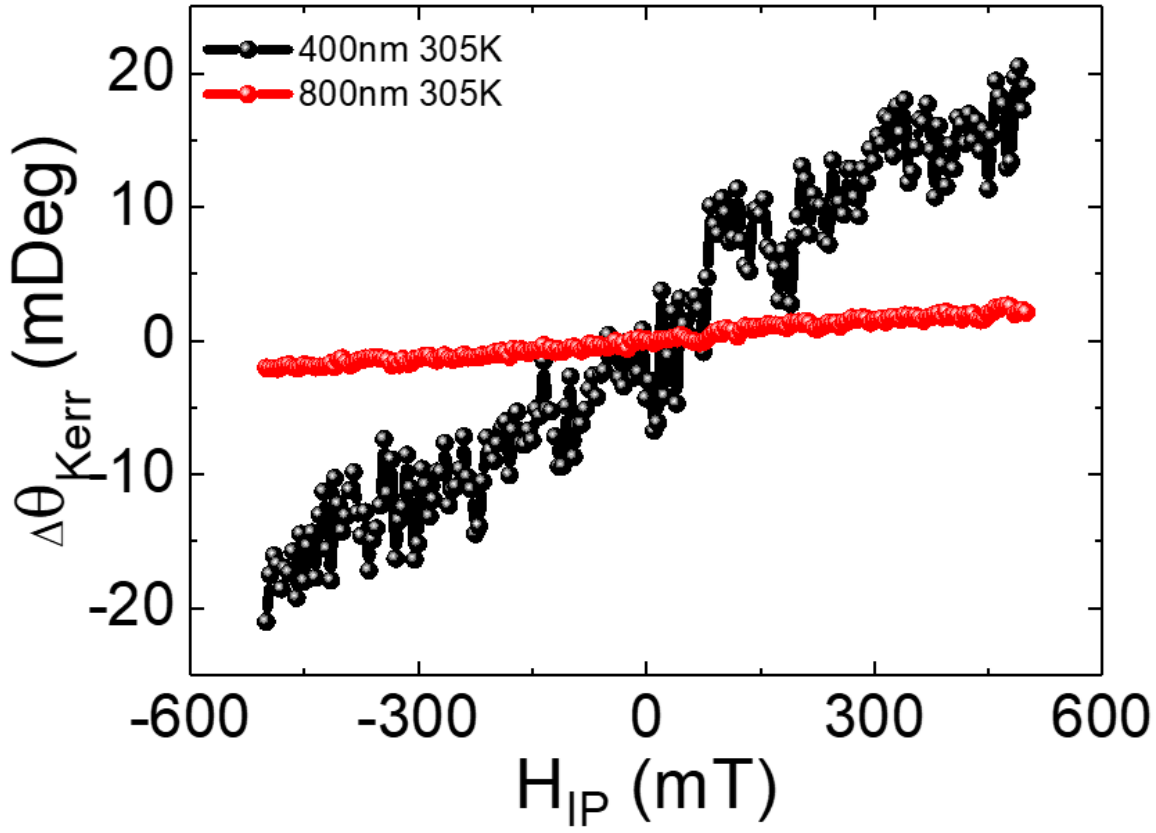

Fig. S5. In-plane Kerr signal as a function of external in-plane magnetic field for λ = 400 nm (black) and λ = 800 nm (red), measured by static-MOKE.

In this work, we found that the magnetic moment at deeper energy level is more susceptible to the magnetic field than that at Fermi level. As we discussed in the main text, the energy-level-selective optical pump-probe measurements in Fig. 3 corroborate the Hall and VSM measurement in Fig. 2. However, the Fig. 3 shows the results in non-equilibrium regime, while that of Fig. 2 is in quasi-static regime. This different dynamic regime between two measurements may cause the possible wrong interpretation. To resolve this issue, we performed the *static*-MOKE measurement which probes the quasi-static properties, like the Hall and VSM measurements.

Figure S5 shows the variation of in-plane static-Kerr signal while sweeping in-plane magnetic field for λ = 400 nm (black) and λ = 800 nm (red) (sweep rates were the same for both measurements). The

measurement setup is identical to that in Fig. 3a of the manuscript. The in-plane static-Kerr signal was obtained by extracting the odd component, as we explained in the manuscript. As can be seen, the Kerr signal for λ = 400 nm is approximately 8 times larger than that for λ = 800 nm. This means that the magnetic moment at deeper energy level is more susceptible to the in-plane magnetic field even at quasi-static regime, which is consistent with the TR-MOKE results in Fig. 3 of manuscript. We note that the larger noise for λ = 400 nm originates from the wave-length-dependent optical sensitivity of our detector, as shown in Fig. S6 (In this study, we used DET36A which is pre-improved version of DET36A2).

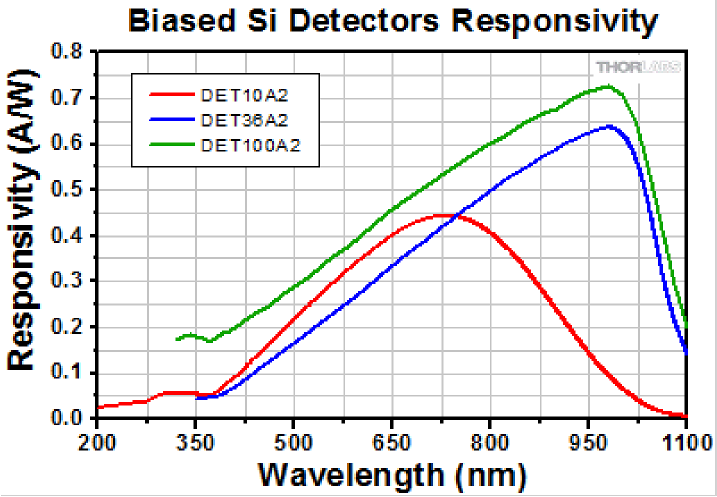

Fig. S6. Wavelength dependent sensitivity of optical detector.

Note 6. Polar MOKE results of TbCo/Pt with 400-nm and 800-nm laser excitation at room temperature

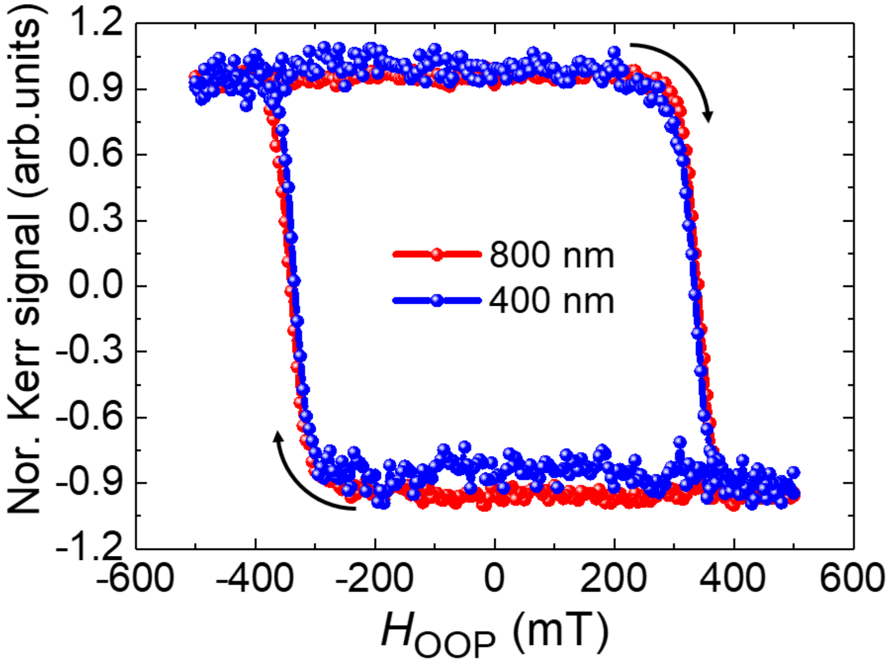

Fig. S7. Normalized polar MOKE results at two different wavelengths. The MOKE signals with 400 and 800 nm laser both show same clock-wise rotation behaviour.

The 4*f* level of Tb in TbCo alloy may be accessible by laser excitation at 400 nm, which can be confirmed by the sign change in magneto-optical Kerr effect (MOKE) [2, 3]. This is because the Tb and Co moments are aligned to antiparallel direction in TbCo alloy, and the effect of Tb moment is increasingly dominant as the laser wavelength moves to a deeper energy level. We are able to confirm whether the MOKE polarity is reversed in our TbCo alloy at shorter wavelength. Our MOKE result of TbCo film in Fig. S7 shows the same polarity for 400 and 800 nm, which suggests that the wavelength of 400 nm might be insufficient to probe the Tb 4*f*-band in our TbCo alloy. The different characteristics between the previous report [3] and ours might be due to different degree of inter-mixing between RE and TM in two samples, because the energy level of the Tb 4f $^{8}S_{7/2}$ state could be changed due to inter-mixing [4]. From these results, it is considered that the maximum density of state of Tb 4*f*-band of our sample may be below 3.1 eV (λ = 400 nm).

Note 7. Quasi-static measurement of slow spin dynamics in TbCo

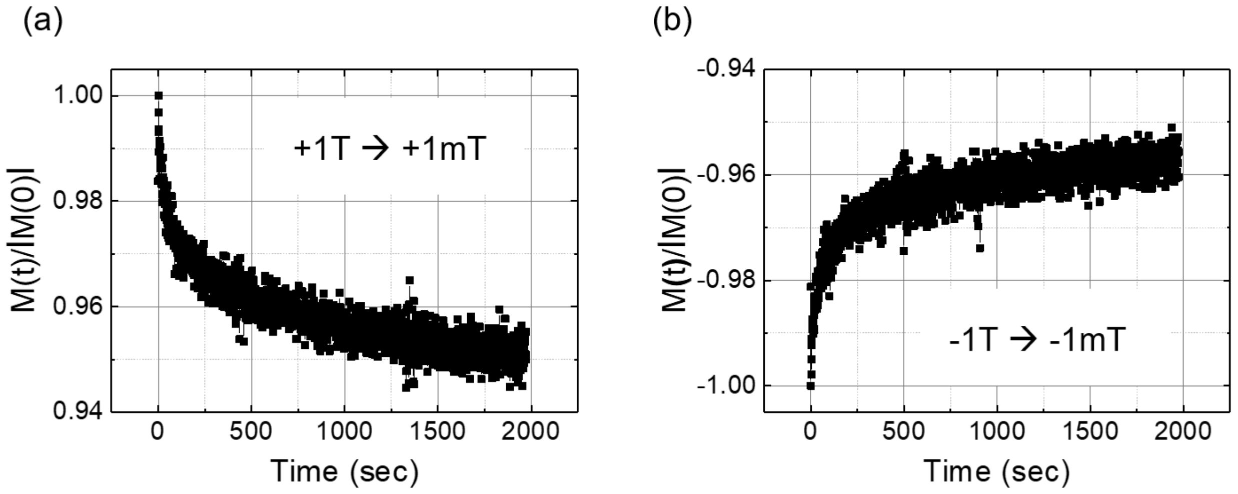

Fig. S8. Magnetic moment as a function of time measured by MPMS for (a) positive field and (b) negative field.

Figure S8 shows the temporal evolution of magnetic moment obtained from TbCo film. The measurement was performed by using MPMS (magnetic properties measurement system). In Fig. S8(a), we first applied magnetic field of H = +1 T along the in-plane direction and subsequently reduced the magnetic field to H = + 1 mT. After that, we measured the magnetic moment in time. $|M(0)|$ is the absolute value of magnetization of the TbCo film immediately after the magnetic field being reduced to +1 mT, and $M(t)$ corresponds to the magnetization variation in time t thereafter. As can be seen, the total magnetic moment decreases slowly over a long time, which indicates the slow relaxation of magnetic moment. The same trend is observed in experiment with opposite magnetic field (Fig. S8(b)). The experiment with opposite magnetic field (Fig. S8(b)) confirms that the slow relaxation is not caused by possible drift in the setup. These *quasi-static* experiments confirm the spin-glass-like slow spin dynamics that we have observed with TR-MOKE measurement.

Note 8. THz wave emission through inverse spin Hall effect

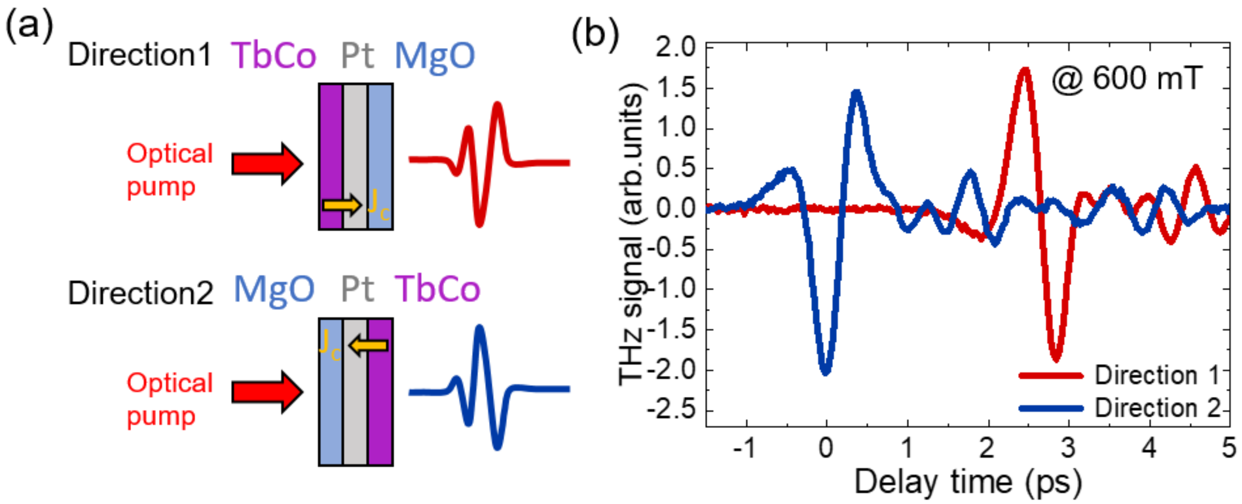

Fig. S9. (a) Schematic diagram of direction of spin current and polarity of generated THz waves generated via inverse spin Hall effect. The flipped sample generates opposite THz polarity, because the order of TbCo and Pt is reversed. (b) Generated THz waves according to sample direction while applying in-plane magnetic field of +600 mT at 800 nm.

The polarity of THz waves emitted via inverse spin Hall effect is affected by the direction of injected spin currents and the external magnetic fields [5]. Accordingly, the polarity of generated THz waves depends on the sample direction and the external magnetic field direction, as shown in Fig. S9 (b) above and Fig. 4(b) of main text, respectively. Note that the delay time difference of THz waves in Fig. S9 (b) originates from two different directions, leading to difference in refractive index of MgO substrate in THz and near-IR range.

Note 9. THz Emission and VSM results for in-plane moment

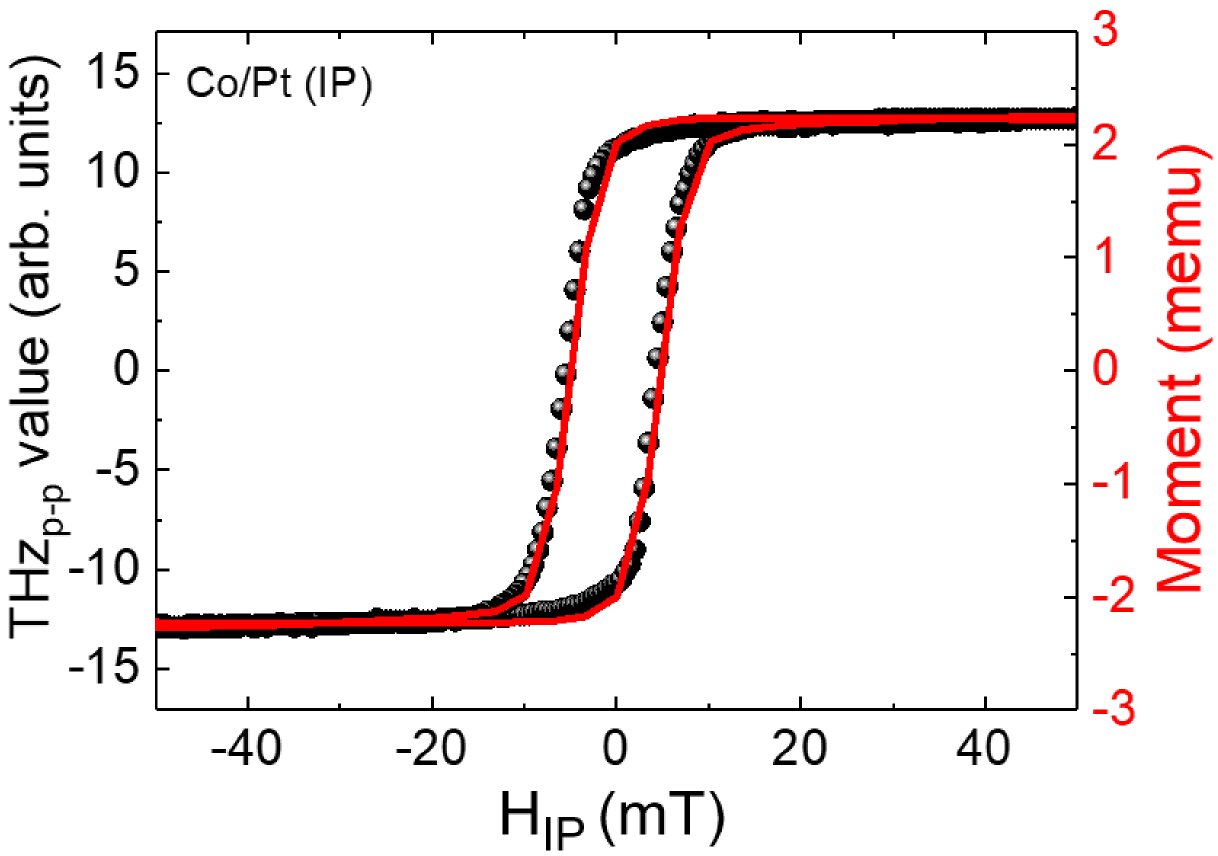

Fig. S10. THz emission and VSM results for Co/Pt film under in-plane field sweeping.

To confirm that THz emission results are related to the in-plane magnetic moment of the sample, we compare THz emission and VSM results for Co/Pt film (SiN (5 nm)/Co (5 nm)/Pt (5 nm) on MgO substrate) having the in-plane magnetic anisotropy. The THz signal is fixed at peak value and recorded while sweeping the in-plane field at room temperature with 800-nm pump. Figure S10 shows that the results of VSM and THz emission exhibit similar trend having same coercive field while sweeping the in-plane field, confirming that the THz emission is indeed sensitive to the in-plane magnetic moment [6].

Note 10. Raw THz emission signals from TbCo/Pt and Co/Pt after field cooling

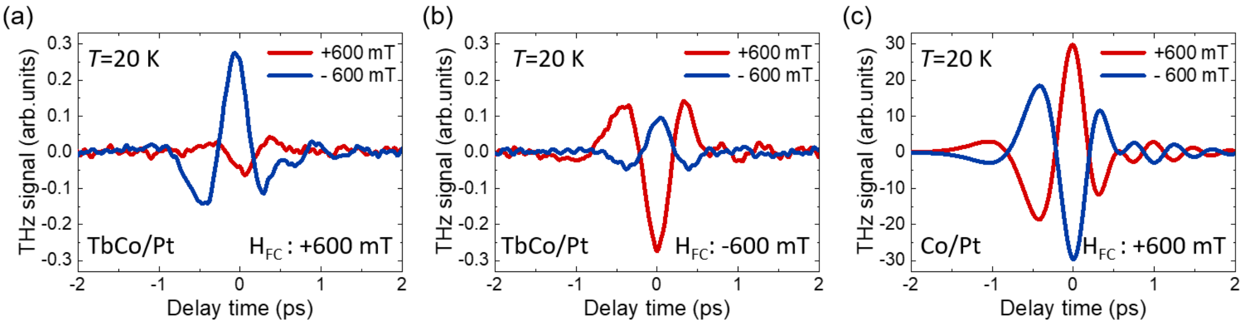

Fig. S11. Measured THz time traces with in-plane fields of $\pm$600 mT after field cooling for (a, b) TbCo/Pt and (c) Co/Pt at 20 K.

Note 11. Measurement of random anisotropy of Tb

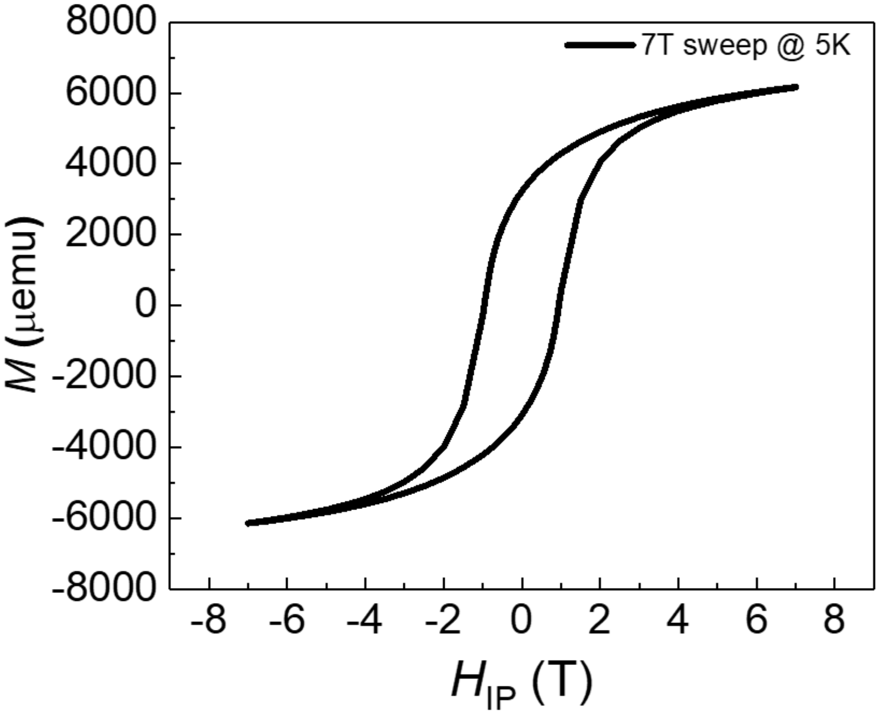

Fig. S12. M-H curve of Si/SiO2// Tb(250nm)/Ta(1.5 nm) sample at 5K.

It is well known that the rare earth Tb has a strong random anisotropy due to the anisotropic 4*f* orbital[7]. To confirm that the our Tb indeed show a strong random anisotropy, we prepared the Tb-only sample by using same DC magnetron sputtering procedure [exact structure is Si/SiO_2/Tb(250 nm)/Ta(1.5 nm); here Ta capping was used for preventing oxidation], and measured the field-dependent magnetization variation. Figure S12 shows raw data of M-H curve for Tb-only sample at T = 5 K. As can be seen, the magnetic moment does not saturate even for H = 7 T which is the maximum magnetic field in the measurement setup (Magnetic Properties Measurement System, MPMS). We note that the positive slope does not originate from the background of substrate, because the diamagnetic Si/SiO_2 substrates produces the negative slope of background signal. The non-saturating positive slope in M-H curve indicates that the anisotropy of randomly deposited Tb is much greater than 7 T. We note that the literature value of Tb anisotropy is much bigger than this, as stated on page 220 of ref. 7. According to ref. 7, order of a million to ten million oersteds is needed to saturate Tb magnetization along the c direction. Considering that our Tb has sputter-deposited

polycrystalline structure, it is reasonable to think that the Tb has a strong random anisotropy field of the magnitude of the order of tens of Tesla. Therefore, the result in Fig. S12 together with the literature suggests that the Tb indeed shows a strong random anisotropy, which could meet the spin-glass condition, as we described in the main text.

Note 12. Spin-glass-like slow dynamics in GdFeCo

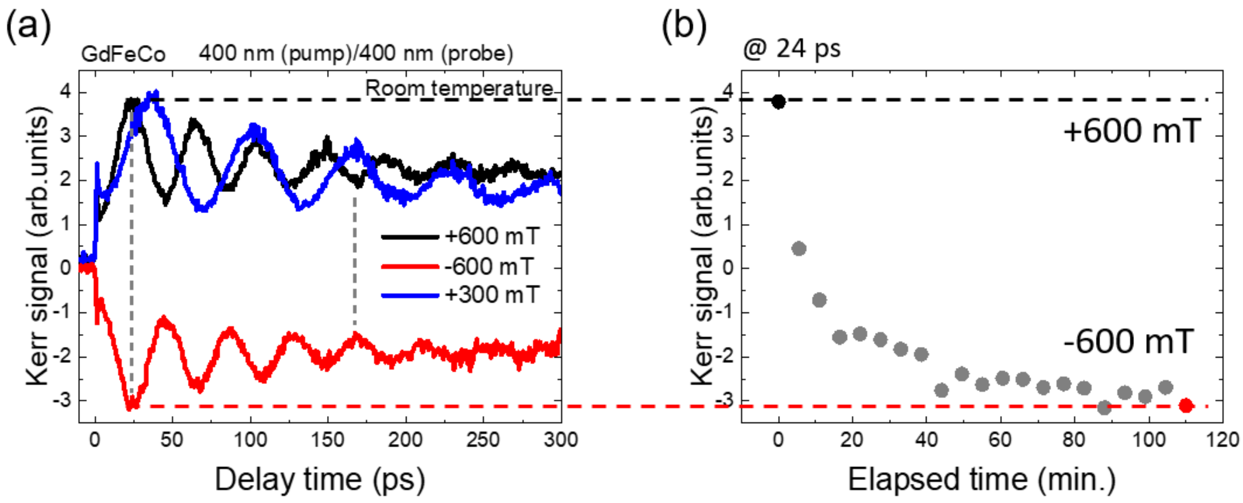

Fig. S13. (a) The 400-nm pump-probe raw data of GdFeCo under external magnetic fields (±600 mT, +300 mT) at room temperature. (b) Slow change of Kerr signal at fixed elapsed time of 24 ps when the external magnetic field is changed from +600 mT to -600 mT.

In this section, we discuss the generality of our finding. In the main text, we used TbCo to demonstrate the energy-level-dependent magnetic moment configuration. The reason why we chose TbCo is that 4*f*-shell of Tb lies in the range of a few eV below E_F that is accessible by visible light [3]. This is a unique advantage of Tb because 4*f*-shell of other rare earths lies in relatively deeper energy level that typically is inaccessible by visible light [8, 9]. In addition, the Tb shows sperimagnetic spin configuration [10, 11]. These characteristics of Tb allow us to discover the unique properties of TbCo, that is, the spin-glass-like properties.

As our approach can be applied to the metallic ferrimagnetic alloys, another candidate would be Gd-based ferrimagnetic alloys, such as GdFe or GdFeCo, as it is another representative material in ferrimagnetic spintronics [12]. Although the 4*f*-shell of Gd lies in much deeper than that of Tb [8], which brings a difficulty to access by visible light, the spin-glass-like slow dynamics can be confirmed in Gd-based ferrimagnet. This is because the GdFeCo exhibits the sperimagnetic behavior owing to the weak exchange interaction of Fe and antiferromagnetic coupling between Fe and Gd, which was demonstrated in our recent paper [13].

Figure S13(a) shows the TR-MOKE measurement result of GdFeCo film. The transient Kerr signal was obtained after exciting and probing the sample using ultrafast pulses at λ =400 nm. The damped oscillation signal is clearly observed for H = + 600 mT (black), indicating the magnetization precession after laser-induced demagnetization. The oscillation period changes when we change the magnetic field to H = + 300 mT (blue), further confirming that the measured signal originates from the magnetization oscillation [14]. We then switched the magnetic field to the opposite direction, that is., H = - 600 mT (red). Figure S13(b) shows the temporal evolution of the Kerr signal for a fixed delay time of 24 ps. Like the TbCo case, the Kerr signal profile of GdFeCo was not switched instantaneously but slowly reversed over time. This demonstrates that the spin-glass-like behavior is indeed related to the sperimagnetic structure. Therefore, the demonstration of slow dynamics in GdFeCo corroborates that the spin-glass-like characteristic observed in TbCo could be generic in other ferrimagnets.

Note 13. Experimental setup for THz emission spectroscopy, static polar MOKE and TR-MOKE

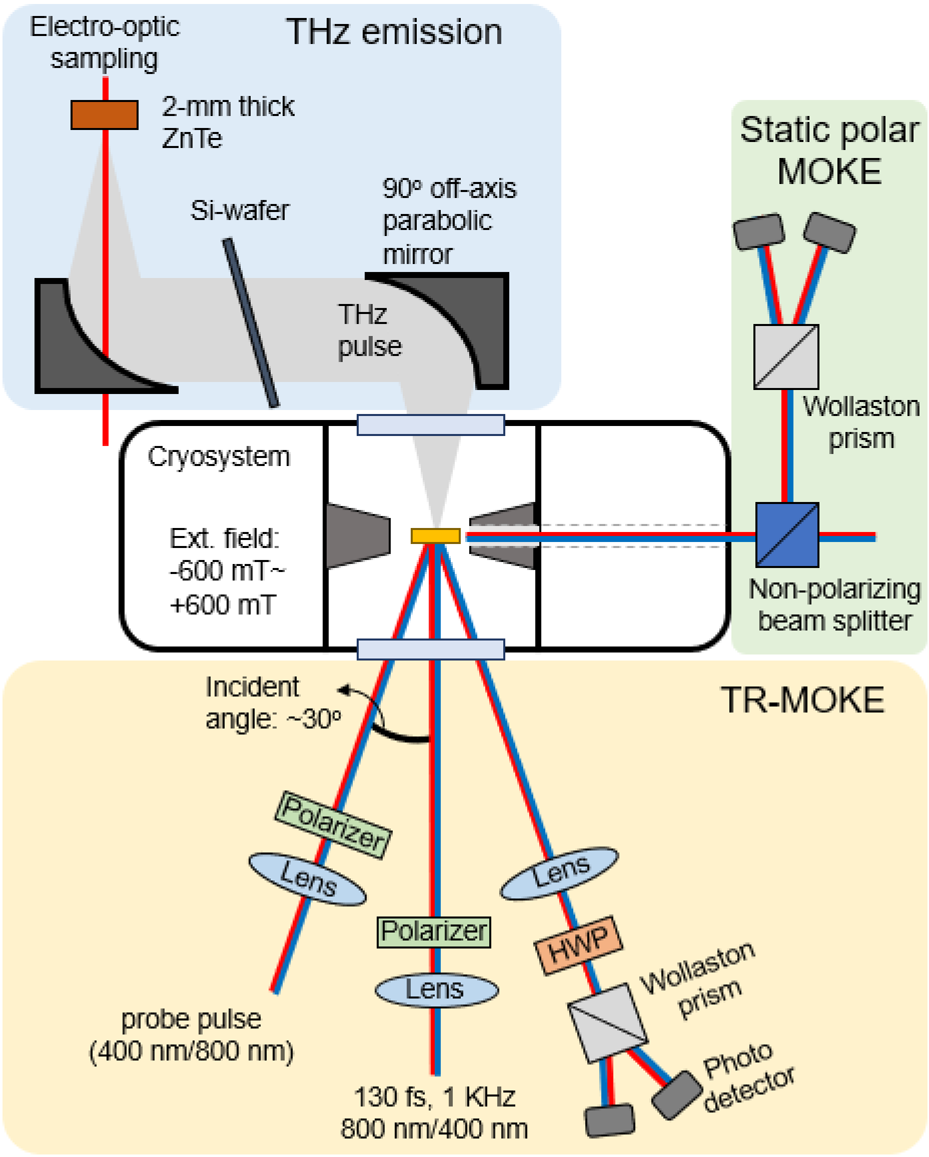

Fig. S14. Schematic diagram of experimental setup for THz emission spectroscopy, static polar MOKE and TR-MOKE.

References

1. Pham, T. H. et al. Thermal contribution to the spin–orbit torque in metallic–ferrimagnetic systems. *Phys. Rev. Appl.* **9**, 064032 (2018)

2. Alebrand, S. et al. Subpicosecond magnetization dynamics in TbCo alloys. *Phys. Rev. B* **89**, 144404 (2014)

3. Khorsand, A. R. et al. Element-specific probing of ultrafast spin dynamics in multisublattice magnets with visible light. *Phys. Rev. Lett.* **110**, 107205 (2013)

4. Melchior P. et al. Energy-resolved magnetic domain imaging in TbCo alloys by valence band photoemission magnetic circular dichroism. *Phys. Rev. B* **88**, 104415 (2013)

5. Qiu, H. S. et al. Layer thickness dependence of the terahertz emission based on spin current in ferromagnetic heterostructures. *Opt. Express* **26**, 15247-15254 (2018)

6. Khusyainov, D. et al. Polarization control of THz emission using spin-reorientation transition in spintronic heterostructure. *Sci. Rep.* **11**, 697 (2021)

7. Wohlfarth, Erich Peter, ed. Handbook of magnetic materials. Vol. 2. Elsevier, (1986)

8. Arenholz, E. *et al.* Magnetic circular dichroism in core-level photoemission from Gd, Tb, and Dy in ferromagnetic materials. *Phys. Rev. B* **51**, 8211 (1995).

9. Thiel, C. W. *et al.* Systematics of 4f electron energies relative to host bands by resonant photoemission of rare-earth ions in aluminum garnets. *Phys. Rev. B* **64**, 085107 (2001).

10. Hassanain. N. et al. Random anisotropy studies in amorphous Co-Tb ribbons. *J. Magn. Magn.Mater.* **140–144**, 337-338 (1995)

11. Yu, J. et al. Topological Hall effect in ferrimagnetic CoTb single layer. *J. Magn. Magn. Mater.* **487**, 165316-7 (2019)

12. Kim, S. K. *et al.,* Ferrimagnetic spintronics. *Nat. Mater.* **21**, 24 (2022).

13. Park, J. *et al*., Unconventional magnetoresistance induced by sperimagnetism in GdFeCo. *Phys. Rev. B* **103**, 014421 (2021).

14. Goncalves, C. S. *et al.,* A Dual-Colour Architecture for Pump-Probe Spectroscopy of Ultrafast Magnetization Dynamics in the Sub-10-femtosecond Range. *Sci. Rep.* **6**, 22872 (2016).
